# Five-Year Changes in Inflammatory, Metabolic, and Oxidative Biomarkers and 10-Year Cardiovascular Disease Incidence: The REGICOR Cohort Study

**DOI:** 10.3390/ijms24097934

**Published:** 2023-04-27

**Authors:** Anna Camps-Vilaro, Isaac Subirana, Rafel Ramos, Miguel Cainzos-Achirica, Helena Tizon-Marcos, Montse Fito, Irene R. Degano, Jaume Marrugat

**Affiliations:** 1 REGICOR Study Group, Hospital del Mar Medical Research Institute (IMIM), 08003 Barcelona, Spain; 2 CIBER of Cardiovascular Diseases (CIBERCV), Instituto de Salud Carlos III (ISCIII), 28029 Madrid, Spain; 3 Doctoral College, University of Vic-Central University of Catalonia (UVic-UCC), 08500 Vic, Spain; 4 Medical Science Department, School of Medicine, University of Girona, 17003 Girona, Spain; 5 Vascular Health Research Group, Institut Universitari per a la Recerca en Atenció Primària Jordi Gol i Gurina, 17002 Girona, Spain; 6 Girona Biomedical Research Institute, 17190 Girona, Spain; 7 Primary Care Services, Catalan Institute of Health, 17005 Girona, Spain; 8 Biomedical Research in Heart Diseases Group, Hospital del Mar Medical Research Institute (IMIM), 08003 Barcelona, Spain; 9 Department of Cardiology, Hospital del Mar, 08003 Barcelona, Spain; 10 Cardiovascular Risk and Nutrition Research Group, Hospital del Mar Medical Research Institute (IMIM), 08003 Barcelona, Spain; 11 CIBER of Pathophysiology of Obesity and Nutrition (CIBEROBN), Instituto de Salud Carlos III (ISCIII), 28029 Madrid, Spain; 12 Faculty of Medicine, University of Vic-Central University of Catalonia (UVic-UCC), 08500 Vic, Spain

**Keywords:** atherosclerosis, biomarkers, cardiovascular diseases, incidence, inflammation, interleukin-6, insulin, coronary artery disease, stroke, peripheral artery disease

## Abstract

Ischemic cardiovascular diseases (CVD) originate from an imbalance between atherosclerotic plaque formation, instability, and endothelial healing dynamics. Our aim was to examine the relationship between 5-year changes in inflammatory, metabolic, and oxidative biomarkers and 10-year CVD incidence in a population without previous CVD. This was a prospective cohort study of individuals aged 35–74 years (n = 419) randomly selected from 5263 REGICOR participants without CVD recruited in 2005. Biomarkers were measured at baseline and in 2010. Participants were followed up until 2020 for a composite CVD endpoint including coronary artery disease, stroke, and peripheral artery disease. We used Cox regression to analyze the effect of biomarker levels on the occurrence of the composite endpoint, adjusted for traditional CVD risk factors and baseline levels of each biomarker. Individuals with elevated IL-6 or insulin after 5 years had a higher independent risk of CVD at 10 years, compared to those with lower levels. Each rise of 1 pg/mL of IL-6 or 10 pg/mL of insulin increased the 10-year risk of a CVD event by 32% and 2%, respectively. Compared to a model with traditional CVD risk factors only, the inclusion of IL-6 and insulin improved continuous reclassification by 51%. Elevated serum levels of IL-6 and insulin were associated with a higher risk of CVD at 10 years, independently of traditional CVD risk factors.

## 1. Introduction

Cardiovascular diseases (CVD), including coronary artery disease and cerebrovascular disease, are the primary cause of mortality and morbidity worldwide, being responsible for approximately 18 million deaths annually, or 32% of all deaths. In European countries, CVD accounts for 3.9 million deaths, or 45% of all deaths [1,2]. In recent decades, more effective treatments, improved patient management, and health promotion initiatives by public health administrations have helped to reduce CVD incidence and mortality in developed countries, including Europe [2,3].

However, more accurate methods are needed to identify those individuals who will have a future CVD event. Currently, high-risk individuals are identified with CVD risk functions; in Europe, these include SCORE2 [4], QRISK [5] and Framingham-REGICOR [6], among others. These functions use baseline data of traditional CVD risk factors and comorbidities to predict the 10-year risk of CVD events. While very useful to organize primary prevention, these CVD risk functions fail to identify all individuals who will develop a CVD event, in part because they do not take into account key atherosclerosis mechanisms.

The origin of ischemic CVD, which accounts for most CVD events, is due to an imbalance between atherosclerotic plaque formation, instability, and endothelial healing dynamics. The first stage of atherosclerosis is described as the modification of subendothelial cells with the retention of lipids [7,8]. Once these lipids have accumulated, oxidative enzymes and oxygen radicals oxidize low-density lipoproteins (LDL) and other lipids that produce changes in the homeostasis of the artery wall and initiate the inflammatory response [9,10]. The actions of endogenous antioxidant enzymes such as glutathione peroxidase (GHS-Px) protect against free radicals to maintain oxidative homeostasis [11]. This oxidative activity causes an inflammatory response from activated endothelial cells and monocytes in the subendothelial space. These activated cells release proinflammatory cytokines such as tumour necrosis factor alpha (TNF-α) and type 1 monocyte chemoattractant protein (MCP)-1, thereby promoting the regulation of migration and infiltration of monocytes/macrophages. Other acute phase inflammatory proteins such as interleukin (IL)-6 and C-reactive protein (CRP) contribute to this inflammatory response [8,12,13,14].

Atherosclerosis is related to chronic inflammation [12,15,16], which destabilizes atherosclerotic plaques. In this regard, IL-6 has been related to plaque erosion and rupture [13], while IL-10 seems to be a key factor in the plaque healing process [17]. Additionally, metabolism biomarkers such as adiponectin correlate with a lower risk of CVD events, likely by inhibiting the action of TNF-α on endothelial cells. High concentrations of biomarkers related to glucose metabolism, such as leptin or insulin, are associated with the development of CVD [18,19]. Oxidative, inflammation, and metabolism biomarkers could be good candidates to predict CVD events, predominantly ischemic stroke, stable angina, unstable angina, and acute myocardial infarction (AMI) [14,18,20]. Although these mechanisms are known in the pathophysiology of atherosclerosis, limited data are available on whether analysis of changes in their plasma levels over time could improve the prediction of future CVD events.

This study had two aims: (1) to analyze the association of 5-year changes and most recent available values in biomarkers of inflammation (TNF-α, IL-10, IL-6, MCP-1, and CRP), metabolism (adiponectin, leptin, and insulin), and oxidation (GHS-Px) with the 10-year incidence of CVD events in a population without previous CVD history, and (2) to examine whether the inclusion of these biomarkers in a coronary risk function improves its discrimination and reclassification capacity.

## 2. Results

**Figure 1:**
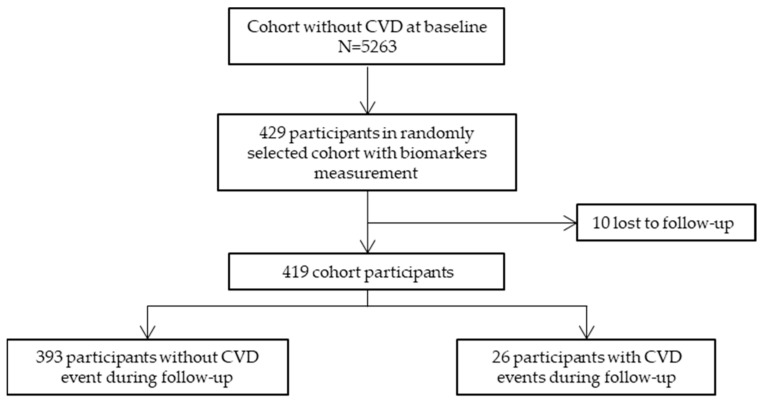
Flowchart of the study participants. CVD, cardiovascular disease.

From the 5263 REGICOR 2005 participants aged 35–74 without previous CVD at baseline, 429 were randomly selected for biomarkers examinations and 419 were followed-up and available for the analysis. Of the included participants, 26 (6.2%) had a reported CVD event during the follow-up (eight coronary artery diseases, eight cerebrovascular diseases, nine peripheral artery diseases, 1 CVD death). A participation flowchart is provided in Figure 1.

Differences in CVD risk factor prevalence between participants with (cases) and without CVD events during the follow-up are shown in Table 1. Cases were 10 years older, more frequently men, and had a significantly higher prevalence of diabetes and hypertension and higher BMI, and systolic and diastolic blood pressure values, compared to participants without CVD events in the follow-up. The proportions of individuals treated for hypertension and dyslipidemia were also higher among cases.

Table 2 shows the 5-year change and the last available value for each biomarker in cases and in participants without CVD events in the follow-up. The 5-year change in IL-10 and insulin differed between groups: cases had a larger increase in the concentration of these two biomarkers, compared with participants with no CVD events. In the most recent available values, cases had higher levels of inflammation biomarkers (TNF-α and IL-6) and the glucidic biomarker (insulin).

Table 3 presents the crude and adjusted hazard ratio (HR) for CVD incidence according to an increase of 1 or 10 units in these biomarkers. Compared to individuals with stable IL-6 or insulin levels, those with increased values after 5 years were at a higher 10-year risk of a CVD event. In addition, after adjusting for potential confounders, increased IL-6 and insulin during the 5-year follow-up were independently associated with 10-year CVD events. A rise of 1 pg/mL and one standard deviation of IL-6 increased by 32% and 54%, respectively, the 10-year risk of developing a CVD event and each rise of 10 pg/mL and one standard deviation of insulin increased the risk by 2% and 56%, respectively. Similar results were obtained when the last available biomarker value was used instead of the 5-year change. Additionally, the effect of one/ten units and one standard deviation, mutually adjusted, of both biomarkers was associated with the incidence of CVD at 10 years with the same adjustment strategy. The remaining biomarkers were not associated with CVD incidence in any model. 

The inclusion of IL-6 and insulin in a model with traditional CVD risk factors significantly improved continuous and categorical reclassification by 51% and 38%, respectively, when compared to the model without biomarkers. Discrimination improved from 65% to 74% but the change did not reach statistical significance (Table 4).

## 3. Discussion

In a population-based cohort, we measured nine biomarkers of inflammation, metabolism, and oxidation (adiponectin, GHS-Px, CRP, insulin, IL-6, IL-10, leptin, MCP-1 and TNF-α). Of those, both the 5-year change and the follow-up levels of insulin and IL-6 were independently associated with a 10-year incidence of fatal and non-fatal CVD events regardless of traditional risk factors. In addition, the most recent available values were enough to predict this association and incorporating either those recent values or the 5-year change in insulin and IL-6 into the model achieved similar improvement in the reclassification obtained by a classical coronary risk model.

Although participants with CVD had a higher prevalence of risk factors, their baseline levels of total and LDL-cholesterol were similar to those without CVD. However, participants with CVD events had lower levels of high-density lipoprotein (HDL)-cholesterol at baseline. Additionally, cases were more frequently treated for hypercholesterolemia. We expected higher total and LDL-cholesterol, and lower HDL-cholesterol in participants with CVD events compared to participants without CVD events in the follow-up. While HDL-cholesterol levels behaved as expected, total and LDL-cholesterol were similar in both groups. This is probably related to the much higher rate of hypercholesterolemia treatment in participants with CVD events.

Elevated levels of insulin have been related to future CVD events in previous studies. Genetic studies have described the causal role of insulin in CVD diseases and metabolic studies suggest that prolonged high levels of insulin and concomitant insulin resistance are accelerators of CVD diseases [21,22]. In addition, baseline levels of insulin have been associated with atherogenic progression [23] and coronary artery disease incidence [18] in population-based cohort studies. Rohling et al. recently reported a significant association between increased fasting insulin over time and the incidence of increased intima media thickness (IMT) and atherosclerosis as precursors of atherogenic progression [23]. Furthermore, two large-scale epidemiological studies showed a significant association between quartiles of insulin resistance in homeostasis model assessment and the incidence of peripheral artery disease [24,25]. In contrast, a meta-analysis involving data from 19 prospective studies in Western populations concludes that the relation between insulin and CVD is weaker than previously suspected. The results have some limitations, especially due to measurement bias, and are based on published grouped data rather than on individual data [19]. However, the role of insulin in the prediction of CVD remains unclear and there is a need for more large-scale and long-term follow-up studies to draw clear conclusions [19,26].

We also found that elevated levels of IL-6 were associated with the incidence of CVD events. IL-6 has emerged as an important cytokine-mediated inflammation with a key role in atherothrombosis [27]. Genetic studies broadly show a causal association between IL-6 and the development of coronary artery disease [28]. Similarly, population-based studies describe a relationship between high levels of IL-6 and the long-term incidence of coronary artery disease [18,29,30,31], stroke [31,32], and peripheral artery disease [31]. On the other hand, median levels of IL-6 in cases and participants without CVD events in our study were similar to the levels detected in men who did or did not develop myocardial infarction in a large prospective study: 1.81 versus 1.46 pg/mL (*p* = 0.002) and 2.21 versus 1.49 pg/mL (*p* = 0.003), respectively [29]. A meta-analysis by Kaptoge et al. showed similar hazard ratios per standard deviation in adjusted models (1.27 vs. 1.32) [30].

Nevertheless, we did not find any association between other prominent biomarkers of inflammation and CVD incidence. Parrinello et al. found that sustained elevated levels of CRP over 6 years are associated with an increased risk of coronary artery disease, ischemic stroke, and mortality [33]. Hassan et al. described that baseline and follow-up levels of TNF type 1 receptor are associated with future CVD mortality in adjusted models. These authors also reported that the follow-up value rather than its change from baseline better predicts mortality [34]. This difference between our results and those obtained by the studies in the United States [33] and Germany [34] could be due to differences in the study population, as those regions have higher CVD risk than our population in northeastern Spain. In addition, the sample size in both studies is larger than ours; our study might not have had sufficient power to detect all significant biomarkers. 

A limited number of studies have tested simultaneously the predictive capacity of the biomarkers analyzed in this study [18,35,36,37]. A systematic review found inconsistent results for CRP [35], one of the few serum biomarkers that some guidelines recommend as a CVD risk modifier [38]. Another study analyzed a baseline measurement of a set of 13 inflammation-related biomarkers, including CRP, IL-6, MCP-1, adiponectin, and leptin, and reported a significant but modest increase in the prediction of coronary events [36]. A third study analyzed a set of biomarkers of inflammation, including CRP and IL-6, and observed a modest predictive capacity of these markers individually [37]. Our results confirmed previous findings of reclassification improvement when insulin is incorporated into a model with traditional CVD risk factors [18]. In addition, our study suggests that reclassification is further improved with the inclusion of insulin and IL-6 in the model.

Furthermore, we found evidence of the role of high levels of insulin and IL-6 in the development of CVD and its importance for future therapeutic targets. According to the CANTOS study, reducing inflammation via IL-1b and IL-6 pathways reduces recurrent CVD events [39]. Ongoing studies with specific IL-6 inhibitors have yielded initial results showing a marked reduction of inflammation and thrombosis biomarkers [40]. Despite these promising reports, the European Society of Cardiology guidelines on cardiovascular prevention recommend against the routine use of biomarkers because improvements in risk prediction tend to be small and publication bias could be an issue [4]. On the other hand, American guidelines on primary prevention of CVD do recommend CRP, lipoprotein a, and apolipoprotein as risk modifiers [38]. Our study was not able to associate CRP with the incidence of CVD, one of the reasons could be the sample size of our study.

While the use of insulin and IL-6 have the potential to provide valuable insights into the underlying mechanisms of CVD, caution should be exercised in their interpretation and application, they should be viewed as just one component of a comprehensive approach. The role of these biomarkers in clinical decision-making should be considered carefully in the context of the individual patient’s characteristics and other relevant clinical factors. To advance our understanding of the role of these biomarkers in predicting CVD, it is imperative to conduct future research utilizing large cohort studies. Such studies can provide valuable insights into the potential utility of these biomarkers and their significance in CVD prediction.

Our study had several strengths. First, participants were from the well-characterized REGICOR population cohort (randomly taken from a total population of 700,000 inhabitants). The REGICOR cohorts have been used to examine coronary artery risk prediction in several studies. Second, we analyzed two measurements made 5 years apart of biomarkers related to the atherosclerosis process and all biomarkers were assayed in the same analytical run, thereby reducing variability and increasing comparability. Third, the study had a robust design that allowed the calculation of discrimination and reclassification statistics.

On the other hand, some limitations must be acknowledged. First, the sample size was sufficient but not large, given budget limitations for analyzing these biomarkers in the entire cohort. However, the cohort was randomly selected and, although modest due to the cost of laboratory testing, the sample had the sufficient statistical power to achieve the objectives defined in the study. In addition, we used a combined endpoint but could not analyze individual events due to a lack of statistical power. Second, it is possible that some biomarkers not included in our study may also play an important role in the development of atherosclerosis and in the prediction of CVD risk. This study was focused on the most solid and recent findings of biomarkers that encompass three biological pathways involved and related to each other in the development of atherosclerosis.

## 4. Materials and Methods

### 4.1. Study Design and Participants

We designed a cohort study nested in the REGICOR cohort recruited in 2005 (5263 participants without CVD) in north-eastern Spain through the Central Register of Users of the Catalan Health Service [41]. Study participants were randomly selected (no stratification and no matching) from the REGICOR 2005 cohort, which included participants aged 35 to 74 years and free of CVD (coronary artery disease, cerebrovascular disease or peripheral artery disease), randomly selected from the population of Girona city and surrounding towns. Only participants with sufficient baseline and follow-up samples were included.

### 4.2. Study Sample Size

A sample size of 400 participants, assuming a 2.5% 10-year CVD incidence and 5% type I error, yielded 80% statistical power to identify as statistically significant a difference greater than or equal to 0.80 standard deviations of a biomarker level between the group with CVD events in the follow-up (n = 9) and the group without CVD events (n = 390).

### 4.3. Re-Examination, Follow-Up, and Composite Endpoint

**Figure 2:**
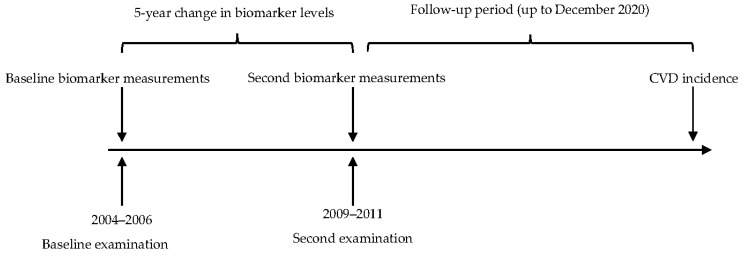
Timeline of the study. The measurement of biomarkers included the following: tumour necrosis factor alpha (TNF-α), interleukin (IL)-6, IL-10, type 1 monocyte attractant protein (MCP-1), C-reactive protein (CRP), glutathione peroxidase (GSH-Px), adiponectin, leptin, and insulin. CVD, cardiovascular disease.

Participants were re-examined in 2009–2011 (Figure 2); they also completed questionnaires regarding lifestyles and CVD risk factors and provided biological samples. Until 2009, the follow-up was carried out by structured telephone interviews, and by data linkage with the AMI REGICOR Registry, hospital discharge records in the Girona region, and the mortality register of Catalonia. From 2009 to 2020, follow-up was performed by data linkage with the hospital discharges, primary care, and mortality data (Data Analysis Program for Health Research and Innovation (PADRIS) from the Health Department of the Catalan government).

The composite endpoint included the fatal and non-fatal first occurrence of coronary artery disease (International Classification of Diseases (ICD)-9 codes: 410–414 and ICD-10 codes: I21-I25, including subtypes), cerebrovascular disease (ICD-9 codes: 430–437 and ICD-10 codes: I60-I69, including subtypes), and peripheral artery disease (ICD-9 codes: 440–443 and ICD-10 codes: I70-I73).

### 4.4. Variables and Laboratory Results

We collected data on demographic and risk factor variables obtained from participants in the REGICOR cohort at inclusion in 2005 and at the 2010 follow-up. The measurements and the administration of questionnaires were carried out by professionals hired and trained for this purpose. Systolic and diastolic blood pressure, body mass index (BMI), smoking status, diabetes, hypertension history, and hypertension and dyslipidemia treatment were obtained by standardized and validated methods [41].

Blood samples were collected after 10–14 h fasting. Serum aliquots were frozen at −80 °C until assayed. Baseline serum glucose, total cholesterol, and triglycerides were measured enzymatically (Roche Diagnostics, Basel, Switzerland) and HDL-cholesterol by a direct methodology in a Cobas Mira Plus autoanalyzer (Roche Diagnostics, Basel, Switzerland). LDL-cholesterol was estimated by the Friedewald equation when triglycerides were lower than 300 mg/dL.

Taking into account the positive results of previous studies [18,30,31,34,35], we analyzed the following biomarkers measured on two occasions 5 years apart (i.e., at inclusion in 2005 and at the follow-up exam in 2010): TNF-α, IL-6, IL-10, MCP-1, CRP, GSH-Px, adiponectin, leptin, and insulin.

GSH-Px levels were determined using cumene hydroperoxide to oxidize glutathione (Ransel RS 505, Randox Laboratories, Crumlin, UK). High-sensitivity CRP was determined by immunoturbidimetry (ABX Diagnostic, Montpellier, France). All other biomarkers were measured using Luminex^®^ xMAP^®^ technology, which is based on the principles of flow cytometry combined with the quantification of elements protected by fluoroimmunoassay; each sample was analyzed using two long-light emission sources that allowed the simultaneous detection and quantification of various combinations of parameters.

### 4.5. Statistical Analysis

Continuous variables following a normal distribution were described as mean and standard deviation and compared using Student’s t-test. For non-normally distributed variables, median and quartiles were used for variable description and Mann–Whitney tests for comparison. Normality was checked graphically and with the Shapiro–Wilk test. Categorical variables were described as numbers and proportions and compared using the Chi-square test. When necessary, biomarker levels that did not follow a normal distribution were log-transformed.

Cox proportional hazards regression was used to model the time to the composite outcome and to estimate the HR for the effect of increasing the level of each biomarker by 1 unit. We assessed the individual effect of each biomarker, adjusted for risk factors (age, sex, systolic and diastolic blood pressure, HDL-cholesterol, total cholesterol, diabetes, smoking, and baseline biomarker levels) and also mutually adjusted.

The biomarkers associated with CVD incidence independently of CVD risk factors were included in a model with traditional CVD risk factors. Improvement in discrimination and reclassification of the model, with and without the significant biomarkers, was calculated by the increment of the C-statistic and the Net Reclassification Index (NRI) (categorical and continuous), respectively. For the categorical NRI, the cut-off points used for 10-year CVD risk were <5% for low-risk, 5 to <10% for intermediate risk and ≥10% for high risk.

Statistical significance was assumed when *p* values were <0.05. All analyses were performed with the R software version 4.1.1, the survival (v3.4-0), and the compareGroups (v4.6.0) R packages [42].

## 5. Conclusions

Our study showed an independent association between the 10-year incidence of CVD events and the 5-year change in insulin and IL-6 levels, after adjustment for traditional CVD risk factors and significant biomarkers. Analysis using the most recent available value yielded similar results and significantly improved reclassification, compared to classical risk functions alone. This study provides evidence for the importance of inflammation and glucose metabolism in atherosclerosis progression and CVD prevention.

## Figures and Tables

**Table 1:** Baseline characteristics of participants with (cases) and without CVD events at 10 years of follow-up.

	Participants without CVD Events in the Follow-Up N = 393	Participants with CVD Events in the Follow-Up (Cases) N = 26	*p* Value
Age, years ^1^	52 (10)	62 (8)	<0.001
Sex, women	56.5%	38.5%	0.112
Smoker	18.7%	26.9%	0.441
Diabetes	10.2%	34.6%	0.001
Glycaemia, mg/dL ^1^	95 (19)	110 (38)	0.060
Glycaemia, treated	3.97%	12.0%	0.093
Hypertension	38.0%	61.5%	0.030
Hypertension, treated	10.7%	30.8%	0.007
Systolic blood pressure, mmHg ^1^	122 (18)	135 (18)	0.001
Diastolic blood pressure, mmHg ^1^	78 (10)	84 (8)	0.004
Hypercholesterolemia, treated	14.8%	44.4%	0.004
Total cholesterol, mg/dL ^1^	212 (44)	213 (55)	0.936
LDL-cholesterol, mg/dL ^1^	138 (39)	136 (38)	0.784
HDL-cholesterol, mg/dL ^1^	54 (13)	49 (12)	0.036
Triglycerides, mg/dL ^2^	89 [66;124]	104 [81;120]	0.066
Body mass index, kg/m^2 1^	26.9 (4.48)	29.1 (4.25)	0.016

^1^ Mean (Standard Deviation); ^2^ Median [interquartile range]. CVD, cardiovascular disease; LDL, low-density lipoprotein; HDL, high-density lipoprotein.

**Table 2:** Difference in the 5-year change and last value of biomarkers in participants with (cases) and without CVD events at 10-year follow-up.

	Participants without CVD Events in the Follow-Up N = 393	Participants with CVD Events in the Follow-Up (Cases) N = 26	*p* Value
MCP-1 (pg/mL) ^1^			
5-year change	−1.00 [−46.8;46.5]	−4.87 [−55.4;60.3]	0.948
Last value	315 [243;396]	352 [301;399]	0.306
TNF-α (pg/mL) ^1^			
5-year change	−0.07 [−0.45;0.30]	0.07 [−0.66;0.54]	0.705
Last value	0.62 [0.17;1.34]	1.29 [0.66;2.11]	0.003
IL-10 (pg/mL) ^1^			
5-year change	−0.05 [−0.32;0.19]	0.04 [−0.08;0.41]	0.007
Last value	0.39 [0.19;0.65]	0.40 [0.14;1.07]	0.805
IL-6 (pg/mL) ^1^			
5-year change	0.00 [−0.42;0.47]	0.16 [−0.31;1.73]	0.100
Last value	1.49 [0.96;2.31]	2.21 [1.49;3.13]	0.003
CRP (mg/dL) ^1^			
5-year change	0.00 [−0.08;0.06]	0.00 [−0.06;0.12]	0.314
Last value	0.10 [0.04;0.24]	0.15 [0.06;0.31]	0.214
Adiponectin (µg/mL) ^1^			
5-year change	0.29 [−0.74;1.29]	0.58 [−0.32;1.28]	0.606
Last value	5.57 [3.55;8.21]	4.58 [2.91;5.90]	0.103
Leptin (ng/mL) ^1^			
5-year change	1.33 [−0.63;4.97]	1.85 [0.04;5.03]	0.580
Last value	8.32 [4.28;13.5]	6.72 [4.96;16.3]	0.759
Insulin (pg/mL) ^1^			
5-year change	28.7 [−55.7;131]	77.6 [18.5;221]	0.029
Last value	235 [135;421]	295 [236;467]	0.021
GHS-Px (U/L) ^1^			
5-year change	−23.0 [−62.0;21.8]	−18.0 [−78.5;10.2]	0.858
Last value	689 [623;761]	648 [607;732]	0.104

^1^ Median [interquartile range]. GHS-Px, glutathione peroxidase; CRP, C-reactive protein; CVD, cardiovascular disease; IL, interleukin; MCP-1, monocyte chemoattractant protein-1; TNF-α, tumour necrosis factor alpha.

**Table 3:** Hazard ratios [and 95% confidence intervals] of cardiovascular disease incidence for one unit of the considered biomarkers after adjustment for traditional cardiovascular risk factors.

	Non-Adjusted		Adjusted for Age, Sex, Baseline Levels & Risk Factors ^1^	
	HR (95%CI)	*p* Value	HR (95%CI)	*p* Value
MCP-1 ^2^				
5-year change	1.00 (0.96–1.04)	0.916	1.01 (0.96–1.05)	0.795
Last value	1.00 (0.98–1.03)	0.785		
TNF-α				
5-year change	1.11 (0.68–1.83)	0.666	1.17 (0.75–1.83)	0.484
Last value	1.36 (1.06–1.74)	0.016		
Interleukin-10				
5-year change	1.08 (0.96–1.21)	0.190	1.10 (0.96–1.26)	0.159
Last value	1.03 (0.90–1.19)	0.647		
Interleukin-6				
5-year change	1.25 (1.05–1.49)	0.011	1.32 (1.10–1.57)	0.002
Last value	1.33 (1.12–1.57)	0.001		
CRP				
5-year change	2.53 (0.72–8.84)	0.146	2.73 (0.63–11.75)	0.177
Last value	1.71 (0.46–6.29)	0.421		
Adiponectin ^2^				
5-year change	1.00 (1.00–1.00)	0.975	1.00 (1.00–1.00)	0.800
Last value	1.00 (1.00–1.00)	0.124		
Leptin ^2^				
5-year change	1.00 (1.00–1.00)	0.157	1.00 (1.00–1.00)	0.118
Last value	1.00 (1.00–1.00)	0.427		
Insulin ^2^				
5-year change	1.02 (1.01–1.03)	0.002	1.02 (1.01–1.03)	0.002
Last value	1.01 (1.00–1.02)	0.010		
GHS-Px ^2^				
5-year change	0.99 (0.94–1.05)	0.857	0.99 (0.94–1.06)	0.842
Last value	0.97 (0.93–1.01)	0.130		

^1^ Systolic and diastolic blood pressure, high-density lipoprotein-cholesterol, total cholesterol, diabetes, and smoking. ^2^ Hazard ratio per 10 units. CI, confidence interval; GHS-Px, glutathione peroxidase; HR, hazard ratio; CRP, C-reactive protein; MCP-1, monocyte chemoattractant protein-1; TNF-α: tumour necrosis factor alpha.

**Table 4:** Reclassification and discrimination improvement when insulin and interleukin-6 were added in a model with traditional risk factors (systolic and diastolic blood pressure, high-density lipoprotein-cholesterol, total cholesterol, diabetes, and smoking).

**Reclassification**	
Continuous NRI	0.51 (0.09–0.92)
Categorical NRI ^1^	0.38 (0.14–0.62)
**Discrimination (C-index)**	
Without biomarkers	0.65 (0.57–0.74)
With biomarkers	0.74 (0.65–0.83)
Difference *p* value	0.08

^1^ For the categorical NRI, 5% and 10% were used as cut-off points. NRI, Net Reclassification Index.

## Data Availability

The datasets generated during and/or analysed during the current study are available from the corresponding author on reasonable request.

## References

[1] Cardiovascular Diseases. https://www.who.int/health-topics/cardiovascular-diseases/#tab=tab_1.

[2] European Cardiovascular Disease Statistics 2017 Edition. https://www.ehnheart.org/cvd-statistics/cvd-statistics-2017.html.

[3] Dégano I.R., Salomaa V., Veronesi G., Ferriéres J., Kirchberger I., Laks T., Havulinna A.S., Ruidavets J.B., Ferrario M.M., Meisinger C. (2015). Twenty-five-year trends in myocardial infarction attack and mortality rates, and case-fatality, in six European populations. Heart.

[4] Visseren F.L., Mach F., Smulders Y.M., Carballo D., Koskinas K.C., Bäck M., Benetos A., Biffi A., Boavida J.M., Capodanno D. (2021). 2021 ESC Guidelines on cardiovascular disease prevention in clinical practice. Eur. Heart J..

[5] Hippisley-Cox J., Coupland C., Brindle P. (2017). Development and validation of QRISK3 risk prediction algorithms to estimate future risk of cardiovascular disease: Prospective cohort study. BMJ.

[6] Marrugat J., Subirana I., Comín E., Cabezas C., Vila J., Elosua R., Nam B.H., Ramos R., Sala J., Solanas P. (2007). Validity of an adaptation of the Framingham cardiovascular risk function: The VERIFICA study. J. Epidemiol. Community Health.

[7] Kwak B.R., Bäck M., Bochaton-Piallat M.L., Caligiuri G., Daemen M.J., Davies P.F., Hoefer I.E., Holvoet P., Jo H., Krams R. (2014). Biomechanical factors in atherosclerosis: Mechanisms and clinical implications. Eur. Heart J..

[8] Wiklund O., Borén J., Krams R., Bäck M. (2017). Pathogenesis of atherosclerosis: Lipid metabolism. The ESC Textbook of Vascular Biology.

[9] Weber C., Noels H. (2011). Atherosclerosis: Current pathogenesis and therapeutic options. Nat. Med..

[10] Glass C.K., Witztum J.L. (2001). Atherosclerosis: The Road Ahead. Cell.

[11] Lubrano V., Balzan S. (2015). Enzymatic antioxidant system in vascular inflammation and coronary artery disease. World J. Exp. Med..

[12] Zhu Y., Xian X., Wang Z., Bi Y., Chen Q., Han X., Tang D., Chen R. (2018). Research Progress on the Relationship between Atherosclerosis and Inflammation. Biomolecules.

[13] Tousoulis D., Oikonomou E., Economou E.K., Crea F., Kaski J.C. (2016). Inflammatory cytokines in atherosclerosis: Current therapeutic approaches. Eur. Heart J..

[14] Danesh J., Wheeler J.G., Hirschfield G.M., Eda S., Eiriksdottir G., Rumley A., Lowe G.D., Pepys M.B., Gudnason V. (2009). C-Reactive Protein and Other Circulating Markers of Inflammation in the Prediction of Coronary Heart Disease. N. Engl. J. Med..

[15] Lusis A.J. (2000). Atherosclerosis. Nature.

[16] Liberale L., Montecucco F., Tardif J.C., Libby P., Camici G.G. (2020). Inflamm-ageing: The role of inflammation in age-dependent cardiovascular disease. Eur. Heart J..

[17] Vergallo R., Crea F. (2020). Atherosclerotic Plaque Healing. N. Engl. J. Med..

[18] Subirana I., Fitó M., Diaz O., Vila J., Francés A., Delpon E., Sanchis J., Elosua R., Muñoz-Aguayo D., Dégano I.R. (2018). Prediction of coronary disease incidence by biomarkers of inflammation, oxidation, and metabolism. Sci. Rep..

[19] Sarwar N., Sattar N., Gudnason V., Danesh J. (2007). Circulating concentrations of insulin markers and coronary heart disease: A quantitative review of 19 Western prospective studies. Eur. Heart J..

[20] Georgakis M.K., De Lemos J.A., Ayers C., Wang B., Björkbacka H., Pana T.A., Thorand B., Sun C., Fani L., Malik R. (2021). Association of Circulating Monocyte Chemoattractant Protein-1 Levels With Cardiovascular Mortality: A Meta-analysis of Population-Based Studies. JAMA Cardiol..

[21] Tikkanen E., Pirinen M., Sarin A.P., Havulinna A.S., Männistö S., Saltevo J., Lokki M.L., Sinisalo J., Lundqvist A., Jula A. (2016). Genetic support for the causal role of insulin in coronary heart disease. Diabetologia.

[22] Kolb H., Kempf K., Röhling M., Martin S. (2020). Insulin: Too much of a good thing is bad. BMC Med..

[23] Röhling M., Kempf K., Kolb H., Martin T., Schneider M., Martin S. (2021). The Epidemiological Boehringer Ingelheim Employee Study (Part 3): Association of Elevated Fasting Insulin Levels but Not HOMA-IR With Increased Intima Media Thickness and Arteriosclerosis in Middle-Aged Persons. Front. Cardiovasc. Med..

[24] Britton K.A., Mukamal K.J., Ix J.H., Siscovick D.S., Newman A.B., de Boer I.H., Thacker E.L., Biggs M.L., Gaziano J.M., Djoussé L. (2012). Insulin resistance and incident peripheral artery disease in the Cardiovascular Health Study. Vasc. Med..

[25] Echouffo-Tcheugui J.B., Chen H., Kalyani R.R., Sims M., Simpson S., Effoe V.S., Correa A., Bertoni A.G., Golden S.H. (2019). Glycemic Markers and Subclinical Cardiovascular Disease: The Jackson Heart Study. Circ. Cardiovasc. Imaging.

[26] Jandeleit-Dahm K.A.M., Gray S.P. (2012). Insulin and cardiovascular disease: Biomarker or association?. Diabetologia.

[27] Libby P., Rocha V.Z. (2018). All roads lead to IL-6: A central hub of cardiometabolic signaling. Int. J. Cardiol..

[28] Swerdlow D.I., Holmes M.V., Kuchenbaecker K.B., Shah T., Stewart M., Lowe G., Nalls M.A., Chung C., Peasey A., Dunlop M. (2012). The interleukin-6 receptor as a target for prevention of coronary heart disease: A mendelian randomisation analysis. Lancet.

[29] Ridker P.M., Rifai N., Stampfer M.J., Hennekens C.H. (2000). Plasma Concentration of Interleukin-6 and the Risk of Future Myocardial Infarction Among Apparently Healthy Men. Circulation.

[30] Kaptoge S., Seshasai S.R.K., Gao P., Freitag D.F., Butterworth A.S., Borglykke A., Di Angelantonio E., Gudnason V., Rumley A., Lowe G.D. (2014). Inflammatory cytokines and risk of coronary heart disease: New prospective study and updated meta-analysis. Eur. Heart J..

[31] Cesari M., Penninx B.W., Newman A.B., Kritchevsky S.B., Nicklas B.J., Sutton-Tyrrell K., Tracy R.P., Rubin S.M., Harris T.B., Pahor M. (2003). Inflammatory markers and cardiovascular disease (The Health, Aging and Body Composition [Health ABC] Study). Am. J. Cardiol..

[32] Papadopoulos A., Palaiopanos K., Björkbacka H., Peters A., de Lemos J.A., Seshadri S., Dichgans M., Marios K. (2022). Circulating Interleukin-6 Levels and Incident Ischemic Stroke. Neurology.

[33] Parrinello C.M., Lutsey P.L., Ballantyne C.M., Folsom A.R., Pankow J.S., Selvin E. (2015). Six-year change in high-sensitivity C-reactive protein and risk of diabetes, cardiovascular disease, and mortality. Am. Heart J..

[34] Hassan L., Medenwald D., Tiller D., Kluttig A., Ludwig-Kraus B., Kraus F.B., Greiser K.H., Mikolajczyk R. (2020). The association between change of soluble tumor necrosis factor receptor R1 (sTNF-R1) measurements and cardiovascular and all-cause mortality-Results from the population-based (Cardiovascular Disease, Living and Ageing in Halle) CARLA study 2002–2016. PLoS ONE.

[35] Lin J.S., Evans C.V., Johnson E., Redmond N., Coppola E.L., Smith N. (2018). Nontraditional Risk Factors in Cardiovascular Disease Risk Assessment: Updated Evidence Report and Systematic Review for the US Preventive Services Task Force. JAMA.

[36] Herder C., Baumert J., Zierer A., Roden M., Meisinger C., Karakas M., Chambless L., Rathmann W., Peters A., Koenig W. (2011). Immunological and Cardiometabolic Risk Factors in the Prediction of Type 2 Diabetes and Coronary Events: MONICA/KORA Augsburg Case-Cohort Study. PLoS ONE.

[37] Wennberg P., Wensley F., Di Angelantonio E., Johansson L., Boman K., Rumley A., Lowe G., Hallmans G., Danesh J., Jansson J.H. (2012). Haemostatic and inflammatory markers are independently associated with myocardial infarction in men and women. Thromb. Res..

[38] Arnett D.K., Blumenthal R.S., Albert M.A., Buroker A.B., Goldberger Z.D., Hahn E.J., Himmelfarb C.D., Khera A., Lloyd-Jones D., McEvoy J.W. (2019). 2019 ACC/AHA Guideline on the Primary Prevention of Cardiovascular Disease: Executive Summary: A Report of the American College of Cardiology/American Heart Association Task Force on Clinical Practice Guidelines. J. Am. Coll. Cardiol..

[39] Ridker P.M., Everett B.M., Thuren T., MacFadyen J.G., Chang W.H., Ballantyne C., Fonseca F., Nicolau J., Koenig W., Anker S.D. (2017). Antiinflammatory Therapy with Canakinumab for Atherosclerotic Disease. N. Engl. J. Med..

[40] Ridker P.M., Devalaraja M., Baeres F.M., Engelmann M.D., Hovingh G.K., Ivkovic M., Lo L., Kling D., Pergola P., Raj D. (2021). IL-6 inhibition with ziltivekimab in patients at high atherosclerotic risk (RESCUE): A double-blind, randomised, placebo-controlled, phase 2 trial. Lancet.

[41] Grau M., Subirana I., Elosua R., Solanas P., Ramos R., Masia R., Cordon F., Sala J., Juvinya D., Cerezo C. (2007). Trends in cardiovascular risk factor prevalence (1995–2000–2005) in northeastern Spain. Eur. J. Cardiovasc. Prev. Rehabil..

[42] R Core Team (2022). R: A Language and Environment for Statistical Computing.

